# 2-Phenyl-5-(trifluoro­meth­yl)pyrazol-3(2*H*)-one

**DOI:** 10.1107/S1600536809029419

**Published:** 2009-07-29

**Authors:** Hugo Gallardo, Edivandro Girotto, Adailton J. Bortoluzzi, Geovana G. Terra

**Affiliations:** a Depto. de Química, Universidade Federal de Santa Catarina, 88040-900 Florianópolis, SC, Brazil

## Abstract

The title compound, C_10_H_7_F_3_N_2_O, is an analogue of pyrazolone derivatives with potential analgesic and anti-inflammatory properties. Its mol­ecular structure consists of phenyl and pyrazol-3(2*H*)-one units with a dihedral angle between the mean planes of the rings of 33.0 (1)°. The crystal structure is stabilized by an inter­molecular hydrogen bond between the N—H group and the carbonyl O atom of the pyrazol-3(2*H*)-one ring which links the mol­ecules into supra­molecular *C*(5) chains along [001] and by weak π–π stacking inter­actions between the phenyl rings [centroid-centroid distance = 3.881 (2) Å]. The F atoms are disordered over two positions with refined site occupancies of 0.768(11) and 0.232(11).

## Related literature

For the analgesic properties of pyrazolones, see: Mehlisch (1983 ▶); Schnitzer (2003 ▶). For the biological activity of some pyrazolone derivatives, see: Pavlov *et al.* (1998 ▶). For the pharmacological properties of pyrazolone deriavtives, see: Kees *et al.* (1996 ▶). For related structures, see: Belmar *et al.* (2006*a* ▶,*b* ▶); Pérez *et al.* (2005 ▶). For metal complexes, see: Hyun-Shin *et al.* (2008 ▶); Gallardo *et al.* (2004 ▶); Meyer *et al.* (1998 ▶). For the synthesis of pyrazolones, see: Nakagawa *et al.* (2006 ▶); Belmar *et al.* (2001 ▶); Bartulín *et al.* (1994 ▶). For hydrogen-bond motifs, see: Bernstein *et al.* (1995 ▶).
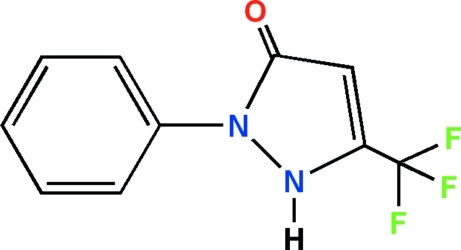


## Experimental

### 

#### Crystal data

C_10_H_7_F_3_N_2_O
*M* *_r_* = 228.18
Monoclinic, 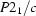

*a* = 5.8409 (5) Å
*b* = 15.2454 (14) Å
*c* = 11.2291 (17) Å
β = 92.403 (9)°
*V* = 999.0 (2) Å^3^
*Z* = 4
Mo *K*α radiation
μ = 0.14 mm^−1^
*T* = 293 K
0.46 × 0.40 × 0.20 mm

#### Data collection

Enraf–Nonius CAD-4 diffractometer
Absorption correction: none
2262 measured reflections
2157 independent reflections
1141 reflections with *I* > 2σ(*I*)
*R*_int_ = 0.024
3 standard reflections every 200 reflections intensity decay: 1%

#### Refinement

*R*[*F* ^2^ > 2σ(*F* ^2^)] = 0.060
*wR*(*F* ^2^) = 0.188
*S* = 1.03
2157 reflections
173 parameters
81 restraints
H-atom parameters constrained
Δρ_max_ = 0.30 e Å^−3^
Δρ_min_ = −0.32 e Å^−3^

### 

Data collection: *CAD-4 Software* (Enraf–Nonius, 1989 ▶); cell refinement: *SET4* in *CAD-4 Software*; data reduction: *HELENA* (Spek, 1996 ▶); program(s) used to solve structure: *SHELXS97* (Sheldrick, 2008 ▶); program(s) used to refine structure: *SHELXL97* (Sheldrick, 2008 ▶); molecular graphics: *PLATON* (Spek, 2009 ▶); software used to prepare material for publication: *SHELXL97*.

## Supplementary Material

Crystal structure: contains datablocks global, I. DOI: 10.1107/S1600536809029419/bx2225sup1.cif

Structure factors: contains datablocks I. DOI: 10.1107/S1600536809029419/bx2225Isup2.hkl

Additional supplementary materials:  crystallographic information; 3D view; checkCIF report

## Figures and Tables

**Table 1:** Hydrogen-bond geometry (Å, °)

*D*—H⋯*A*	*D*—H	H⋯*A*	*D*⋯*A*	*D*—H⋯*A*
N2—H2⋯O1^i^	0.88	1.89	2.667 (3)	146

Symmetry code: (i) 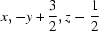.

**Fig. 1.**
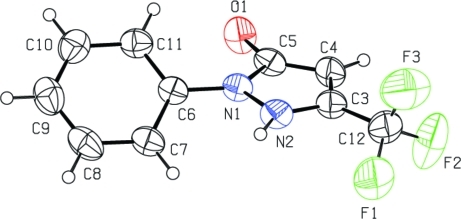
The molecular structure of (I) with labeling scheme. Displacement ellipsoids are shown at the 40% probability level.

**Fig. 2.**
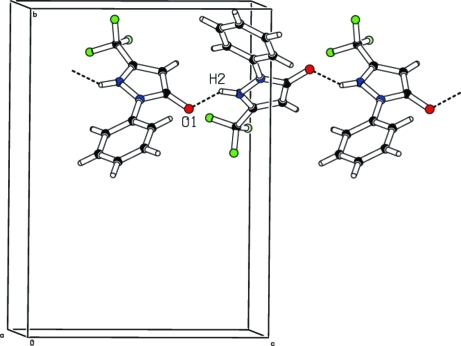
Part of the crystal structure of (I), showing the formation of a C(5) chain pattern. [Symmetry code: (i) x,-y+3/2,z-1/2]

## supplementary crystallographic information

### Comment

The pyrazolone analgesics (such as phenylbutazone) have effects similar to those of aspirin. They were commonly used to treat rheumatoid arthritis and has been the focus of medicinal chemists for over last 100 years because of the outstanding pharmacological properties shown by several of its derivatives (Kees *et al.*, 1996). The interest in such compounds, pyrazolone derivative, arises from the fact that the incorporation of heteroatoms can result an ancillary ligand to study their photoactive lanthanide complexes. These compounds possess several sites for substitution, allowing for a systematic analysis of their effects on the photo optical properties. Particulary the luminescence properties of the Eu and Tb complexes.

The molecular structure of (I) consists of a phenyl group bonded to 2-N of the dihydropyrazole heterocyclic ring (Fig. 1). These rings are twisted with respect to each other and the dihedral angle between the mean plane is 33.0 (1)°.The molecules are linked into chains by one intermolecular N—H···O hydrogen bond. Atoms N2 in the molecules at (x,y,z) acts as hydrogen bonds donor vía atom H2 to atoms O1 at (-x, 3/2+y, -1-z) so generating by translation one C(5) chains running parallel to [001] direction (Bernstein *et al.*, 1995), (Fig. 2, Table 1) and the crystal structure is reinforced by a weak face-to-face π-π stacking interactions between phenyl rings with  the centroid-centroid distance of 3.881 (2) Å.

### Refinement

All non-H atoms were refined with anisotropic displacement parameters. H_Ar_ atoms were placed at their idealized positions with distances of 0.93 Å and *U*_eq_ fixed at 1.2 *U*_iso_ of the preceding atom. H atom attached to N atom was located from Fourier difference map and treated with riding model. Fluorine atoms are disordered over two alternative positions with refined site occupancies of 0.768 (11) and 0.232 (11).

### Crystal data

**Table:**

C_10_H_7_F_3_N_2_O	*F*(000) = 464
*M**_r_* = 228.18	*D*_x_ = 1.517 Mg m^−^^3^
Monoclinic, *P*2_1_/*c*	Melting point = 464–465 K
Hall symbol: -P 2ybc	Mo *K*α radiation, λ = 0.71073 Å
*a* = 5.8409 (5) Å	Cell parameters from 25 reflections
*b* = 15.2454 (14) Å	θ = 3.2–13.8°
*c* = 11.2291 (17) Å	µ = 0.14 mm^−^^1^
β = 92.403 (9)°	*T* = 293 K
*V* = 999.0 (2) Å^3^	Irregular, colourless
*Z* = 4	0.46 × 0.40 × 0.20 mm

### Data collection

**Table:**

Enraf–Nonius CAD-4 diffractometer	*R*_int_ = 0.024
Radiation source: fine-focus sealed tube	θ_max_ = 27.0°, θ_min_ = 2.3°
graphite	*h* = −7→7
ω–2θ scans	*k* = −19→0
2262 measured reflections	*l* = −14→0
2157 independent reflections	3 standard reflections every 200 reflections
1141 reflections with *I* > 2σ(*I*)	intensity decay: 1%

### Refinement

**Table:**

Refinement on *F*^2^	Primary atom site location: structure-invariant direct methods
Least-squares matrix: full	Secondary atom site location: difference Fourier map
*R*[*F*^2^ > 2σ(*F*^2^)] = 0.060	Hydrogen site location: inferred from neighbouring sites
*wR*(*F*^2^) = 0.188	H-atom parameters constrained
*S* = 1.03	*w* = 1/[σ^2^(*F*_o_^2^) + (0.0843*P*)^2^ + 0.3445*P*] where *P* = (*F*_o_^2^ + 2*F*_c_^2^)/3
2157 reflections	(Δ/σ)_max_ = 0.001
173 parameters	Δρ_max_ = 0.30 e Å^−^^3^
81 restraints	Δρ_min_ = −0.32 e Å^−^^3^

### Special details

**Table:**

Experimental. The title compound was synthesized by the condensation of ethyl 4,4,4-trifluoroacetoacetate (5.0?g, 27.2?mmol) in acetic acid (50?ml) with phenylhydrazine (2.9?g, 27.2?mmol) which was added drop wise, with stirring for 3?h. The solvent was removed by evaporation; resulting crude solid was extracted with AcOEt. The organic layer was washed with saturated aqueous NaHCO3 and water, then brine, and evaporation the solvent. The compound, obtained as colorless single crystals, was recrystallized using ethylacetate and n-hexane (2:1) and was suitable for X-ray structure determination. Yield 76% mp: 191–192 °C, lit. 195–196 °C (Nakagawa *et al.*, 2006). 1H-NMR (DMSO, 400?MHz, d, p.p.m.) 12,42 (1H, s), 7,70 (2H, d, J = 8?Hz), 7,49 (2H, t, J = 8?Hz), 7,36 (1H, t, J = 8?Hz), 5,92 (1H, s). 13C-NMR (DMSO, 400?MHz, d, p.p.m.) 153,68 (C5), 140,21 (C3), 13,70 (C6), 129,07 (C10; C8), 127,18 (C9), 122,25 (C11; C7), 119,98 (C12), 85,53 (C4).

### Fractional atomic coordinates and isotropic or equivalent isotropic displacement parameters (Å^2^)

**Table:**

	*x*	*y*	*z*	*U*_iso_*/*U*_eq_	Occ. (<1)
N1	0.2789 (5)	0.78258 (17)	0.98039 (19)	0.0486 (7)	
N2	0.3999 (5)	0.73266 (19)	0.90418 (19)	0.0534 (7)	
H2	0.3729	0.7405	0.8270	0.064*	
C3	0.5425 (6)	0.6863 (2)	0.9721 (3)	0.0535 (8)	
C4	0.5221 (6)	0.7045 (2)	1.0923 (3)	0.0567 (9)	
H4	0.6052	0.6801	1.1566	0.068*	
C5	0.3528 (6)	0.7661 (2)	1.0950 (2)	0.0523 (8)	
C6	0.1101 (5)	0.8436 (2)	0.9370 (2)	0.0471 (8)	
C7	0.1383 (6)	0.8843 (2)	0.8282 (2)	0.0582 (9)	
H7	0.2671	0.8730	0.7847	0.070*	
C8	−0.0280 (7)	0.9420 (3)	0.7854 (3)	0.0730 (11)	
H8	−0.0111	0.9694	0.7123	0.088*	
C9	−0.2193 (7)	0.9594 (3)	0.8499 (4)	0.0745 (11)	
H9	−0.3304	0.9983	0.8204	0.089*	
C10	−0.2439 (6)	0.9189 (3)	0.9575 (3)	0.0691 (10)	
H10	−0.3715	0.9309	1.0016	0.083*	
C11	−0.0805 (6)	0.8604 (2)	1.0010 (3)	0.0583 (9)	
H11	−0.0994	0.8323	1.0735	0.070*	
C12	0.7009 (8)	0.6240 (3)	0.9172 (3)	0.0712 (11)	
F1	0.7641 (13)	0.6502 (4)	0.8115 (4)	0.118 (3)	0.768 (11)
F1'	0.617 (3)	0.5831 (17)	0.826 (2)	0.129 (8)	0.232 (11)
F2	0.8938 (12)	0.6159 (7)	0.9809 (5)	0.129 (3)	0.768 (11)
F2'	0.884 (4)	0.6549 (11)	0.882 (3)	0.141 (9)	0.232 (11)
F3	0.6134 (13)	0.5470 (4)	0.9008 (9)	0.144 (3)	0.768 (11)
F3'	0.760 (5)	0.5604 (14)	0.9906 (13)	0.104 (6)	0.232 (11)
O1	0.2602 (4)	0.81041 (16)	1.18269 (16)	0.0686 (8)	

### Atomic displacement parameters (Å^2^)

**Table:**

	*U*^11^	*U*^22^	*U*^33^	*U*^12^	*U*^13^	*U*^23^
N1	0.0599 (16)	0.0617 (17)	0.0244 (11)	0.0019 (14)	0.0042 (10)	−0.0008 (11)
N2	0.0666 (17)	0.0714 (18)	0.0225 (11)	0.0001 (14)	0.0044 (11)	−0.0034 (11)
C3	0.062 (2)	0.062 (2)	0.0374 (16)	0.0015 (17)	0.0057 (15)	0.0011 (15)
C4	0.068 (2)	0.068 (2)	0.0336 (15)	0.0072 (19)	0.0006 (14)	0.0062 (15)
C5	0.070 (2)	0.063 (2)	0.0242 (14)	−0.0023 (18)	0.0033 (13)	0.0040 (13)
C6	0.0554 (19)	0.0511 (18)	0.0343 (15)	−0.0052 (16)	−0.0026 (13)	−0.0028 (13)
C7	0.072 (2)	0.067 (2)	0.0360 (15)	−0.0042 (19)	0.0003 (15)	0.0052 (15)
C8	0.093 (3)	0.068 (3)	0.056 (2)	−0.011 (2)	−0.015 (2)	0.0164 (18)
C9	0.075 (3)	0.063 (2)	0.083 (3)	0.001 (2)	−0.022 (2)	0.005 (2)
C10	0.059 (2)	0.073 (2)	0.075 (2)	0.001 (2)	−0.0004 (18)	−0.004 (2)
C11	0.062 (2)	0.066 (2)	0.0470 (18)	−0.0056 (19)	0.0017 (16)	0.0011 (16)
C12	0.078 (3)	0.083 (3)	0.054 (2)	0.010 (2)	0.018 (2)	−0.003 (2)
F1	0.142 (6)	0.152 (5)	0.064 (3)	0.052 (4)	0.050 (3)	0.008 (3)
F1'	0.083 (11)	0.172 (19)	0.129 (14)	0.035 (11)	−0.038 (10)	−0.112 (12)
F2	0.100 (4)	0.187 (7)	0.099 (4)	0.068 (4)	−0.016 (3)	−0.030 (4)
F2'	0.105 (13)	0.127 (13)	0.20 (2)	−0.035 (10)	0.083 (14)	−0.071 (15)
F3	0.164 (6)	0.077 (3)	0.197 (8)	−0.008 (3)	0.088 (6)	−0.041 (4)
F3'	0.133 (15)	0.104 (11)	0.074 (8)	0.059 (10)	−0.003 (9)	−0.004 (8)
O1	0.0983 (19)	0.0804 (17)	0.0276 (11)	0.0248 (15)	0.0078 (11)	−0.0044 (10)

### Geometric parameters (Å, °)

**Table:**

N1—C5	1.363 (3)	C8—C9	1.382 (6)
N1—N2	1.365 (3)	C8—H8	0.9300
N1—C6	1.426 (4)	C9—C10	1.371 (5)
N2—C3	1.312 (4)	C9—H9	0.9300
N2—H2	0.8825	C10—C11	1.381 (5)
C3—C4	1.388 (4)	C10—H10	0.9300
C3—C12	1.479 (5)	C11—H11	0.9300
C4—C5	1.366 (5)	C12—F2'	1.247 (14)
C4—H4	0.9300	C12—F1'	1.279 (13)
C5—O1	1.327 (4)	C12—F3	1.291 (7)
C6—C11	1.374 (4)	C12—F3'	1.309 (13)
C6—C7	1.387 (4)	C12—F2	1.315 (6)
C7—C8	1.381 (5)	C12—F1	1.320 (5)
C7—H7	0.9300		
			
C5—N1—N2	109.7 (3)	C9—C8—H8	119.6
C5—N1—C6	129.0 (2)	C10—C9—C8	119.5 (4)
N2—N1—C6	121.2 (2)	C10—C9—H9	120.3
C3—N2—N1	105.6 (2)	C8—C9—H9	120.3
C3—N2—H2	136.6	C9—C10—C11	120.4 (4)
N1—N2—H2	117.7	C9—C10—H10	119.8
N2—C3—C4	112.3 (3)	C11—C10—H10	119.8
N2—C3—C12	119.8 (3)	C6—C11—C10	119.9 (3)
C4—C3—C12	127.9 (3)	C6—C11—H11	120.1
C5—C4—C3	104.5 (3)	C10—C11—H11	120.1
C5—C4—H4	127.7	F2'—C12—F1'	103.5 (9)
C3—C4—H4	127.7	F2'—C12—F3'	105.9 (9)
O1—C5—N1	119.0 (3)	F1'—C12—F3'	103.0 (9)
O1—C5—C4	133.2 (3)	F3—C12—F2	108.5 (5)
N1—C5—C4	107.9 (3)	F3—C12—F1	105.8 (5)
C11—C6—C7	120.4 (3)	F2—C12—F1	104.6 (5)
C11—C6—N1	120.4 (3)	F2'—C12—C3	116.7 (8)
C7—C6—N1	119.2 (3)	F1'—C12—C3	114.9 (7)
C8—C7—C6	118.9 (3)	F3—C12—C3	113.1 (4)
C8—C7—H7	120.5	F3'—C12—C3	111.6 (7)
C6—C7—H7	120.5	F2—C12—C3	111.8 (4)
C7—C8—C9	120.8 (3)	F1—C12—C3	112.5 (4)
C7—C8—H8	119.6		
			
C5—N1—N2—C3	0.6 (4)	C6—C7—C8—C9	0.3 (5)
C6—N1—N2—C3	178.3 (3)	C7—C8—C9—C10	0.0 (6)
N1—N2—C3—C4	−0.6 (4)	C8—C9—C10—C11	−0.7 (6)
N1—N2—C3—C12	179.7 (3)	C7—C6—C11—C10	−0.7 (5)
N2—C3—C4—C5	0.4 (4)	N1—C6—C11—C10	−179.4 (3)
C12—C3—C4—C5	180.0 (4)	C9—C10—C11—C6	1.1 (5)
N2—N1—C5—O1	178.1 (3)	N2—C3—C12—F2'	84.0 (18)
C6—N1—C5—O1	0.6 (5)	C4—C3—C12—F2'	−95.6 (18)
N2—N1—C5—C4	−0.4 (4)	N2—C3—C12—F1'	−37.5 (17)
C6—N1—C5—C4	−177.9 (3)	C4—C3—C12—F1'	143.0 (17)
C3—C4—C5—O1	−178.1 (4)	N2—C3—C12—F3	−88.1 (7)
C3—C4—C5—N1	0.0 (4)	C4—C3—C12—F3	92.3 (7)
C5—N1—C6—C11	−35.3 (5)	N2—C3—C12—F3'	−154.2 (17)
N2—N1—C6—C11	147.5 (3)	C4—C3—C12—F3'	26.3 (18)
C5—N1—C6—C7	146.0 (3)	N2—C3—C12—F2	149.0 (7)
N2—N1—C6—C7	−31.2 (4)	C4—C3—C12—F2	−30.5 (9)
C11—C6—C7—C8	0.0 (5)	N2—C3—C12—F1	31.7 (7)
N1—C6—C7—C8	178.7 (3)	C4—C3—C12—F1	−147.9 (5)

### Hydrogen-bond geometry (Å, °)

**Table:**

*D*—H···*A*	*D*—H	H···*A*	*D*···*A*	*D*—H···*A*
N2—H2···O1^i^	0.88	1.89	2.667 (3)	146

Symmetry codes: (i) *x*, −*y*+3/2, *z*−1/2.
